# Antimicrobial Properties of Plant Essential Oils against Human Pathogens and Their Mode of Action: An Updated Review

**DOI:** 10.1155/2016/3012462

**Published:** 2016-12-20

**Authors:** Mallappa Kumara Swamy, Mohd Sayeed Akhtar, Uma Rani Sinniah

**Affiliations:** ^1^Department of Crop Science, Faculty of Agriculture, Universiti Putra Malaysia, 43400 Serdang, Selangor, Malaysia; ^2^Padmashree Institute of Management and Sciences, Kommagatta, Kengeri, Bangalore 560060, India; ^3^Department of Botany, Gandhi Faiz-E-Aam College, Shahjahanpur, Uttar Pradesh 242001, India

## Abstract

A wide range of medicinal and aromatic plants (MAPs) have been explored for their essential oils in the past few decades. Essential oils are complex volatile compounds, synthesized naturally in different plant parts during the process of secondary metabolism. Essential oils have great potential in the field of biomedicine as they effectively destroy several bacterial, fungal, and viral pathogens. The presence of different types of aldehydes, phenolics, terpenes, and other antimicrobial compounds means that the essential oils are effective against a diverse range of pathogens. The reactivity of essential oil depends upon the nature, composition, and orientation of its functional groups. The aim of this article is to review the antimicrobial potential of essential oils secreted from MAPs and their possible mechanisms of action against human pathogens. This comprehensive review will benefit researchers who wish to explore the potential of essential oils in the development of novel broad-spectrum key molecules against a broad range of drug-resistant pathogenic microbes.

## 1. Introduction

Medicinal and aromatic plants (MAPs) constitute a large part of natural flora and are considered an important resource in various fields such as the pharmaceutical, flavor and fragrance, perfumery, and cosmetic industries [1]. At present, more than 80% of the global population depends on traditional plant-based medications for treating various human health problems [2–4]. According to an estimate, the worth of herbal products on the global market is approximately 62 billion USD, and it is predicted to grow up to 5 trillion USD by the year 2050 [5]. More than 9000 native plants have been identified and recorded for their curative properties, and about 1500 species are known for their aroma and flavor. Essential-oil–based products or natural aroma chemicals are in higher demand in the cosmetic, food, perfume, and pharmaceutical industries, and more than 250 types of essential oils, at a value of 1.2 billion USD, are traded annually on the international market [3, 6].

Essential oils obtained from MAPs are aromatic in nature because of a mixture of multifarious chemical substances that belong to different chemical families, including terpenes, aldehydes, alcohols, esters, phenolic, ethers, and ketones [3, 7]. Essential oils have tremendous business potential on the global market owing to their unique flavor and fragrance properties and also biological activities [6, 8]. Essential oils are employed in aromatherapy and for the treatment of several diseases including cardiovascular disease, diabetes, Alzheimer's, cancer [9]. The antimicrobial impacts of essential oils and their chemical components have been recognized by several researchers in the past [3, 10–13]. Furthermore, studies have shown the synergistic effect of any two or more ingredients of essential oils against various human pathogens [14, 15].

More recently, the prevalence of antimicrobial drug resistance has prompted researchers to discover novel antimicrobial lead molecules to treat various human pathogens [16]. Some of the presently available synthetic drugs fail to inhibit many pathogenic microbes. In addition, the use of synthetic chemicals for the control of pathogenic microorganisms is limited because of their carcinogenic effects, acute toxicity, and environmental hazard potential. In this regard, the exploitation of essential oils to control epidemic multidrug-resistant pathogenic microorganisms can be useful to combat various infectious diseases [17]. Therefore, the present review details the antibacterial, antifungal, and antiviral potentials of essential oils extracted from MAPs as well as their therapeutic relevance and possible mechanisms involved in the reticence of human pathogenic microorganisms. In addition, this review suggests avenues for more research studies on essential oils to be used against drug-resistant microbial pathogens.

## 2. Chemical Composition of Essential Oils

**Figure 1:**
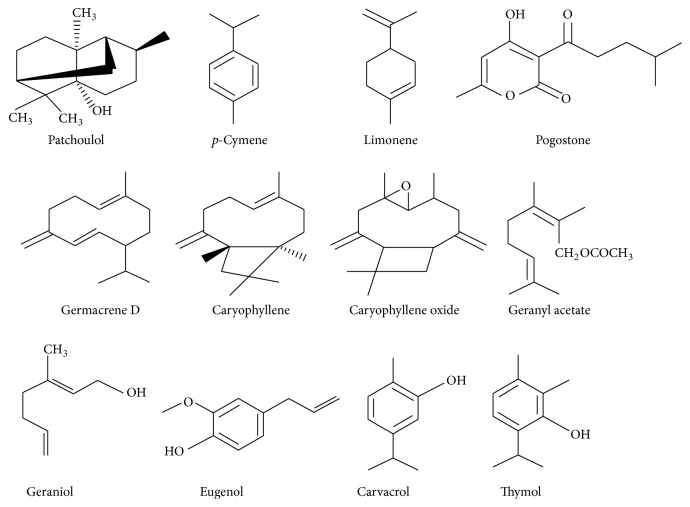
Structures of some important chemical compounds of essential oils.

Essential oils have the ability to hamper the growth of a diverse range of pathogens because of the presence of natural compounds produced by the organs of plants. Importantly, the unique aroma and other bioactive properties of an essential oil depend on its chemical constituents. In MAPs, essential oils generally accumulate in the secretary canals or cavities and glandular trichomes and sometimes in the epidermal cells [4]. Essential oils and their chemical constituents exhibit more bioactivity when present in the oxygenated or active form. In general, the chemical composition of essential oils is relatively complex, and about 20 to 60 different bioactive components are observed in many of these essential oils. Many of these compounds are pharmaceutically appreciated for their numerous culinary properties [1, 4, 7, 13]. Usually, the chemical characterization of many essential oils reveals the presence of only 2-3 major components at a fairly high concentration (20–70%) compared to other components present in trace amounts [101]. Most essential oils are composed of terpenes, terpenoids, and other aromatic and aliphatic constituents with low molecular weights. Terpenes or terpenoids are synthesized within the cytoplasm of the cell through the mevalonic acid pathway [15]. Terpenes are composed of isoprene units and are generally represented by the chemical formula (C_5_H_8_)_*n*_. Terpenes can be acyclic, monocyclic, bicyclic, or tricyclic [102]. Owing to the diversity in their chemical structures, terpenes are classified into several groups such as monoterpenes (C_10_H_16_), sesquiterpenes (C_15_H_24_), diterpenes (C_20_H_32_), and triterpenes (C_30_H_40_). The major component (~90%) of bioactive essential oils is constituted of monoterpenes [103]. Some of the major compounds include monoterpene hydrocarbons (*p*-cymene, limonene, *α*-pinene, and *α*-terpinene), oxygenated monoterpenes (camphor, carvacrol, eugenol, and thymol), diterpenes (cembrene C, kaurene, and camphorene), sesquiterpene hydrocarbons (*β*-caryophyllene, germacrene D, and humulene), oxygenated sesquiterpenes (spathulenol, caryophyllene oxide), monoterpene alcohols (geraniol, linalool, and nerol), sesquiterpene alcohol (patchoulol), aldehydes (citral, cuminal), acids (geranic acid, benzoic acid), ketones (acetophenone, benzophenone), lactones (bergapten), phenols (eugenol, thymol, carvacrol, and catechol), esters (bornyl acetate, ethyl acetate), and coumarins (fumarin, benzofuran) [1, 4, 8, 13, 104, 105]. The structures of some of these compounds are represented in Figure 1. The major and biologically important chemical constituents of MAPs are shown in Tables 1, 2, and 3.

The chemical constituents of plant essential oils differ between species. Some factors that can affect these constituents include the geographical location, environment, and stage of maturity [4, 106]. This chemical difference is directly related to differences in antimicrobial activities against various pathogenic microorganisms [107]. For example, the major chemical constituents of origanum essential oil (carvacrol and thymol) were shown to differ in their origin as well as antimicrobial property.

Furthermore, the stereochemical properties of essential oils can vary and depend upon the method of extraction [108]. However, extraction products may also vary qualitatively and quantitatively in their composition [109]. Although essential oils can be recovered using fermentation, extraction, or effleurage processes, commercial production is preferably achieved by the steam distillation process [1, 4, 110]. Likewise, the antimicrobial efficiency of essential oils depends on the type of microbes to be inhibited as well as the evaluation methods, including bioautography, diffusion, and dilution [111, 112]. Methods to evaluate the essential oil chemistry, their biological activities, and various factors that affect bioactivity are detailed in the literature [110, 112, 113].

## 3. Antimicrobial Effects of Essential Oils

The antimicrobial effects of essential oils derived from MAPs are the basis of copious applications, in various revenue generating sectors such as pharmaceutical, nutraceutical, cosmetic, perfume, agronomy, and sanitary industries [1–3]. In the following section, we have broadly discussed the antibacterial, antifungal, and antiviral effects of essential oils obtained from MAPs.

### 3.1. Antibacterial Effects of Essential Oils

At present, many antibiotics are available for treating various bacterial pathogens. However, increased multidrug resistance has led to the increased severity of diseases caused by bacterial pathogens. In addition, low immunity in host cells and the ability of bacteria to develop biofilm-associated drug resistance have further increased the number of life-threatening bacterial infections in humans [114]. Thus, bacterial infections remain a major causative agent of human death, even today. In addition, the use of several antibacterial agents at higher doses may cause toxicity in humans. This has prompted researchers to explore alternative new key molecules against bacterial strains [115]. In this regard, plant essential oils and their major chemical constituents are potential candidates as antibacterial agents. Several types of essential oils and their major chemical constituents from various MAPs have been reported to possess a wide range of bacterial inhibitory potentials (Table 1).

The effect of antibacterial activity of essential oils may inhibit the growth of bacteria (bacteriostatic) or destroy bacterial cells (bactericidal). Nevertheless, it is difficult to distinguish these actions. In relation to this, antibacterial activity is more frequently measured as the minimum bactericidal concentration (MBC) or the minimum inhibitory concentration (MIC) [110]. Rapid antibacterial screening of essential oils is usually conducted using the agar diffusion technique, where essential oils are added to filter paper discs or holes, which are put in agar that has been uniformly inoculated with a bacterial strain. After incubating, the inhibition zone represents the antimicrobial action [111]. The effectiveness of essential oils differs from one type to another as well as against different target bacteria depending on their structure (Gram-positive and Gram-negative bacteria). For instance, sandalwood and vetiver oils exhibit higher inhibitory activity against Gram-positive bacteria; however, they fail to inhibit Gram-negative bacterial strains [83, 114]. The essential oils of cinnamon, clove, pimento, thyme, oregano, and rosemary were shown to possess strong antibacterial activity against* Salmonella typhi*,* Staphylococcus aureus*, and* Pseudomonas aeruginosa *[116]. Clove oil was found to be the most effective among all the tested essential oils. The antimicrobial effect of these oils was correlated to the occurrence of the major compounds such as carvacrol, thymol, cinnamic aldehyde, eugenol, and* p*-cymene. Likewise, carvacrol, eugenol, and thymol obtained from MAPs have been shown to effectively inhibit food-borne pathogens such as* Escherichia coli*,* Salmonella typhimurium*,* Listeria monocytogenes*, and* Vibrio vulnificus *[117]. The compounds such as benzoic acids, benzaldehydes, and cinnamic acid have shown up to 50% inhibition of* Listeria monocytogenes *under anaerobic conditions [118]. Ouattara et al. [119] reported the antibacterial potential of clove, cinnamon, pimento, and rosemary essential oils against meat spoilage bacterial pathogens such as* Pseudomonas fluorescens*,* Serratia liquefaciens*,* Brochothrix thermosphacta*,* Carnobacterium piscicola*,* Lactobacillus curvatus*, and* Lactobacillus sake*. According to them, the 1/100 dilution of these essential oils was capable of inhibiting at least 5-6 of the tested microbes. The inhibitory effect of these oils was mainly correlated with the occurrence of eugenol and cinnamaldehyde in the essential oils. Other major compounds found were carvacrol, thymol, cinnamaldehyde, and camphor. Arora and Kaur [120] analyzed the antimicrobial activity of garlic, ginger, clove, black pepper, and green chilli on human pathogenic bacteria such as* Bacillus sphaericus*,* Enterobacter aerogenes*,* E. coli*,* Pseudomonas aeruginosa*,* S. aureus*,* Staphylococcus epidermidis*,* S. typhi*, and* Shigella flexneri*. They concluded that, among all these spices, the aqueous extract of garlic was sensitive against all the tested bacterial pathogens. The garlic extract inhibited 93% of* S. epidermidis* and* S. typhi *within 3 h of incubation time. Similarly, the effect of clove extracts on the production of verotoxin by* E. coli *was studied by Sakagami et al. [121], who found that verotoxin production was inhibited by the clove extract (MIC value of >1.0% w/v). The effectiveness of cardamom, anise, basil, coriander, rosemary, parsley, dill, and angelica essential oils against pathogenic and saprophytic microorganisms was examined by Elgayyar et al. [58]. They concluded that essential oils extracted from oregano, basil, and coriander plants have an inhibitory effect against* P. aeruginosa*,* S. aureus*, and* Yersinia enterocolitica* in the range of 400 ppm concentration. Skandamis et al. [122] observed the significance of oregano essential oils on the behavior of* S. typhimurium *in sterile and naturally contaminated beef fillets stored under aerobic and customized atmospheric conditions. The addition of oregano essential oils (0.8% v/w) reduced the majority of the tested bacterial pathogens. Hood et al. [23] reported that the bacterial growth may be suppressed by the ample use of essential oils or their use at high concentrations and that their mode of action results in the decline of bacterial cells. In another study,* Achillea clavennae* essential oil exhibited maximum inhibitory activity against respiratory disease-causing microbes like* Klebsiella pneumoniae*,* Streptococcus pneumoniae*,* Haemophilus influenzae*, and* P. aeruginosa* [18]. The oil contained eucalyptol (1,8-cineole) and camphor as major compounds. According to Nevas et al. [49], pathogenic bacteria such as* Clostridium botulinum and Clostridium perfringens* were effectively inhibited by oregano, savory, and thyme essential oils. The major compounds with an antibacterial effect were found to be camphor, thymol, and carvacrol. The essential oil of* Salvia officinalis* contains *α*-thujone, camphor, and 1,8-cineole as the major chemical constituents and was shown to inhibit human bacterial pathogens such as* S. aureus *and* Providencia stuartii* [67]. Some pathogenic bacteria (*Salmonella choleraesuis*,* Salmonella enteritidis*,* S. typhimurium*, and* E. coli*) were inhibited by the essential oils of thyme and oregano [50]. The essential oils showed an MIC value of 0.25% to ≥2% v/v. In another study,* Salvia* spp. (*S. officinalis*,* S. sclarea*, and* S. lavandulifolia*) and* Thuja* spp. (*T. plicata *and* T. occidentalis*) essential oils exhibited potent antimicrobial properties against human pathogens [68]. The major components (*α*-thujone and *β*-thujone) of these sage species demonstrated high inhibitory activity against* P. aeruginosa* and* K. pneumoniae*, whereas* S. aureus* and* E. coli *were moderately inhibited.

The antibacterial activity of oregano oil against* S. aureus*,* Bacillus subtilis*,* E. coli*, and* P. aeruginosa* was reported by Santoyo et al. [51]. The MBC values ranged between 0.75 and 2.25 mg/mL. Carvacrol was the most effective compound with an MBC value of 0.75 to 1.53 mg/mL, followed by linalool with 1.04 to 1.75 mg/mL. Similarly, oregano essential oil was also shown to be effective against* Providencia stuartii *and* E. coli* [52]. The essential oils of* Thuja* spp. (*T. plicata* and* T. occidentalis*) effectively inhibited* P. aeruginosa*,* K. pneumoniae*,* S. aureus*, and* E. coli *[68]. Moreover, Chaieb et al. [34] revealed the antimicrobial potential of the essential oil of* Eugenia caryophyllata* against numerous multidrug-resistant* S. epidermidis* strains isolated from dialysis biomaterials. Saet et al. [32] reported the presence of* n*-mentha-1,8-dien-10-al, limonene, geranial, and neral as the major constituents in* Dracocephalum foetidum* essential oil. The oil exhibited antibacterial activity against human pathogenic bacteria such as* S. aureus, B. subtilis, Enterococcus hirae*,* E. coli*,* Micrococcus luteus*,* Streptococcus mutans, *and* Saccharomyces cerevisiae*. The MIC value ranged from 26 to 2592 *μ*g/mL. Likewise, Botelho et al. [39] reported the antibacterial activity of* Lippia sidoides *oil against four strains of cariogenic bacteria, namely,* Streptococcus sanguis, S. mutans, Streptococcus salivarius*, and* Streptococcus mitis*. The MIC value ranged from 0.625 to 10.0 mg/mL. Lopes-Lutz et al. [21] reported that several species of* Artemisia* essential oil possessed strong activity against* E. coli*,* S. aureus*, and* S. epidermidis*. Likewise,* Momordica charantia* seed essential oil exhibited inhibitory action against* E. coli* and* S. aureus* with an MIC value of >500 and 125 *μ*g/mL, respectively [44]. The medicinal plant* Achillea ligustica* containing terpinen-4-ol, *β*-pinene, 1,8-cineole, and linalool showed effective inhibitory activity against* S. mutans* with an MIC ranging from 155 to 625 *μ*g/mL [20]. Many food-borne and spoilage bacterial pathogens were inhibited by* Satureja cuneifolia* essential oil and the MIC values were in the range of 600–1400 *μ*g/mL [71]. The essential oil of* Coriandrum sativum* demonstrated an antimicrobial potential against a wide range of bacterial pathogens, but the highest inhibition was found against* Bacillus cereus* and* E. coli*. The MIC of oil for Gram-positive bacteria was observed to be 108 mg/mL and, for Gram-negative bacteria, it ranged from 130 to 217 mg/mL [26]. Moreover, the essential oils extracted from thyme and mint leaves exhibited antibacterial activity against the* S. aureus, S. typhimurium, Vibrio parahaemolyticus, L. monocytogenes, E. coli, C. botulinum, C. perfringens, Shigella sonnei, Sarcina lutea, *and* Micrococcus flavus* [40, 75]. The Gram-negative bacterial strains showed more sensitivity towards the thyme oil. The MIC value ranged from 0.33 to 2.67 mg/mL [75]. The essential oil of* Myrtus communis* was reported to inhibit various bacterial strains such as* S. aureus, L. monocytogenes, Enterococcus durans, S. typhi*,* Enterobacter cloacae, E. coli, B. subtilis, Mycobacterium tuberculosis, P. aeruginosa, K. pneumoniae, *and* Mycobacterium avium *[85, 123]. Similarly, Unlu et al. [24] reported that diverse range of bacterial pathogens such as* S. aureus, Streptococcus pyogenes, S. pneumoniae, Enterococcus faecalis, Enterococcus faecium, B. cereus, Acinetobacter lwoffii, E. aerogenes, E. coli, K. pneumoniae, Proteus mirabilis, P. aeruginosa, S. typhimurium, C. perfringens, *and* Mycobacterium smegmatis *were inhibited by the essential oil of* Cinnamomum zeylancium*. In a study by Shan et al. [78], the essential oils of cinnamon, oregano, clove, pomegranate peels, and grape seeds were found to be effective against* S. enterica*, but the clove extracts possessed the highest antibacterial activity.* Melaleuca alternifolia* (tea tree oil) and its major constituent, terpinen-4-ol, were shown to possess potential antibacterial properties against many pathogens including* E. coli, S. aureus, S. epidermidis, E. faecalis, P. aeruginosa, M. avium, H. influenzae, S. pyogenes, *and* S. pneumoniae*. Overall, it was shown that tea tree oil and terpinen-4-ol have limited influence on the development of antibacterial resistance and susceptibility [42]. Ait-Ouazzou et al. [30] studied the essential oil composition and antibacterial potential of* Mentha pulegium*,* Juniperus phoenicea*, and* Cyperus longus* and concluded that all these oils were effective against food-borne pathogens (*S. aureus, L. monocytogenes, E. faecium, S. Enteritidis, E. coli, *and* P. aeruginosa*). According to them,* M. pulegium* exhibited the best antibacterial activity compared to* J. phoenicea* and* C. longus*. The MIC value of* M. pulegium *oil was <0.5 for* E. faecium* and 1 *μ*L/mL for* S. aureus, L. monocytogenes*,* E. coli,* and* S. enteritidis*. Lawal et al. [124] have reported the antibacterial activity of essential oil of* Ocimum gratissimum, O. kilimandscharicum*,* O. lamiifolium*, and* O. suave* against* S. aureus, Bacillus *sp.,* E. coli, P. aeruginosa, S. typhi, K. pneumoniae, *and* P. mirabilis*. The MIC values varied between 1.25 and 10 mg/mL (flower oil) and between 0.16 and 10 mg/mL (leaf oil). The thyme oil obtained from leaves showed the presence of camphor, camphene, *α*-pinene, 1,8-cineole, borneol, and *β*-pinene, which exhibited effective antibacterial activity against* S. aureus, S. epidermidis, Streptococcus *sp.*, Pantoa *sp., and* E. coli *[53, 76]. The thyme oil showed MIC and MBC values of 627.7 *μ*g/mL and 990.2 *μ*g/mL, respectively, against the* E. coli* strain. The major compound thymol showed MIC and MBC values of 2786 *μ*g/mL and 2540 *μ*g/mL, respectively. Therefore, this study proposes the possible use of thyme oil as a potential antimicrobial agent for food preservation [76]. The oil obtained from* Laurus nobilis* and* Lavandula intermedia *showed inhibitory potential against* Mycobacterium smegmatis *and* E. coli* [37]. The bacterial strains (*Shigella sonnei, Sarcina lutea, *and* Micrococcus flavus*) were inhibited by the essential oil of* Origanum vulgare* [54]. The zone of inhibition and MIC values of* O. vulgare* oil were in the range of 9–36 mm and 125–600 *μ*g/mL, respectively. Several food-borne pathogens such as* Brochothrix thermosphacta, E. coli, Listeria innocua, L. monocytogenes, Pseudomonas putida, S. typhimurium, *and* Shewanella putrefaciens* were inhibited by some commercial essential oils including those of* Ocimum basilicum*,* Petroselinum sativum,* and* Rosmarinus officinalis* [22]. The essential oil of* Syzygium cumini* was found to contain *α*-pinene, *β*-pinene,* trans*-caryophyllene, 1,3,6-octatriene, delta-3-carene, *α*-caryophyllene, and limonene as major chemical compounds and possessed effective antibacterial activity against pathogenic bacterial strains such as* E. coli, S. aureus, P. aeruginosa, Neisseria gonorrhoeae*,* B. subtilis, and S. aureus* [73]. The essential oil exhibited moderate inhibition zones (12–14 mm) against the tested microbes. Andrade et al. [25] studied the antimicrobial activity of 27 different essential oils employed in aromatherapy procedures and found that* Piper nigrum, Melaleuca alternifolia, Copaifera officinalis, *and* Cinnamomum cassia *essential oils were effective against* S. aureus* and* E. coli*, whereas* S. aromaticum* essential oil was efficient against* P. aeruginosa* strains. Khoury et al. [38] have reported that* Juniperus excelsa* essential oil obtained from leaves and twigs was efficient at inhibiting* S. aureus* (MIC value of 64 mg/ml) and* Trichophyton rubrum* (MIC value of 128 mg/mL). Although the essential oil of* Mentha suaveolens* showed strong antibacterial activity against* S. xylosus* with an MIC value of 14.4 *μ*L/mL, it showed no activity against lactic acid bacterial strains except* Lactococcus lactis *[41]. The essential oil of the herb* Struchium sparganophora* revealed the presence of *β*-caryophyllene, germacrene A, *α*-humulene, and germacrene D as major chemical constituents and it exhibited antibacterial activity against* S. typhi, B. cereus, B. subtilis, P. mirabilis, *and* P. aeruginosa *[72]. The inhibitory zone for leaf oil ranged from 9.0 ± 1.0 to 14.3 ± 2.55 mm, whereas the essential oil from stem had inhibitory activity ranging from 18.5 ± 2.2 to 20.0 ± 0.0 mm.* Daucus littoralis* oil obtained from different parts of the plant has showed a strong antibacterial activity against* E. coli *and* S. aureus* with an MIC value ranging from 20 to 40 *μ*L/mL [31]. Likewise, Beatovic et al. [57] have reported the antibacterial activity of* Ocimum basilicum* oil against* S. typhimurium* and* E. coli*. The MIC values ranged between 0.009 and 23.48 *μ*g/mL, whereas the MBC values ranged from 0.28 to 135 *μ*g/mL. In addition, essential oil of Australian-grown* Ocimum tenuiflorum* (Tulsi) showed antibacterial activity against selected microbial pathogens including methicillin-resistant* S. aureus *(MRSA)*, E. coli, *and* P. aeruginosa* with MIC values ranging from 2.25 to >4.5 *μ*g/mL [125]. The essential oil of* Pogostemon cablin *was shown to have effective antibacterial activity against many pathogenic bacterial strains including* E. coli, S. aureus, K. pneumoniae, *and* H. pylori* [1, 60–64]. The GC-MS analysis of essential oils of* Foeniculum vulgare* (Fennel) showed the occurrence of* trans*-anethole, methylchavicol, limonene, and fenchone, whereas* Cuminum cyminum* L. had *γ*-terpin-7-al, *γ*-terpinene, *β*-pinene, and cuminaldehyde as the major constituents. Both essential oils were effective against* S. typhimurium *and* E. coli* [28]. The* F. vulgare* oil exhibited the lowest MIC values of 0.062 and 0.031% (v/v) against* E. coli* and* S. typhimurium,* respectively, whereas* C. cyminum* oil showed MIC values of 0.250 and 0.125% (v/v) against* E. coli* and* S. typhimurium,* respectively. The bacterial strains* S. aureus, B. cereus, *and* P. aeruginosa *were strongly inhibited by the essential oil of* Warionia saharae, *which contained *β*-eudesmol,* trans*-nerolidol, linalool, 1,8-cineole, camphor,* p*-cymene, and terpinen-4-ol as major compounds [79]. The MICs ranged between 0.039 and 0.156 mg/mL for all tested bacterial strains. The essential oil extracted from seeds of* Trachyspermum ammi *showed activity against all 36 clinical isolates of* K. pneumoniae, E. coli, *and* S. aureus *isolated from patients suffering from urinary tract infections [74]. An MIC value of 250 ppm was observed for* K. pneumoniae*, whereas it was observed to be 100 ppm for* E. coli* and* S. aureus*. The seed essential oils of* Nigella sativa* containing thymoquinone,* p*-cymene, *α*-thujene, thymohydroquinone, and longifolene as major phytocompounds were shown to exhibit strong antibacterial activity against* B.  cereus, E.  coli, P. aeruginosa,* and* S.  aureus*. The oil was highly effective against* B. cereus, B. subtilis,* and* S. aureus *and showed a complete zone of inhibition at 3000 ppm concentration. Moreover, the zones of inhibition for* P. aeruginosa* and* E. coli* were 20 and 25 mm, respectively [126]. A study by Cui et al. [69] has shown that* Salvia sclarea *oil showed a considerable inhibitory potential against the growth of* E. coli, S. aureus, Bacillus pumilus, K. pneumoniae, B. subtilis, S. typhimurium, *and* P. aeruginosa* with MIC and MBC of 0.05 and 0.1%, respectively. Ahmadi et al. [77] reported the antibacterial properties of* Thymus kotschyanus* essential oil against* B. cereus*,* E. coli, S. aureus*, and* S. epidermidis. *The MIC values for these pathogens ranged from 0.097 to 6.25 *μ*L/mL. The antibacterial activity of* Euphrasia rostkoviana* essential oil against* E. faecalis*,* E. coli*,* K. pneumoniae*,* S. aureus*,* S. epidermidis*, and* P. aeruginosa* was reported by Novy et al. [35]. In the study, all Gram-positive bacteria were effectively inhibited with an MIC of 512 *μ*g/mL. The bacterial strain* S. epidermidis* was inhibited by the essential oils of* Plectranthus barbatus* and* P. amboinicus* with an MIC value of 31 *μ*g/mL [4, 33]. Likewise, the essential oil of* Plectranthus neochilus* was shown to inhibit some cariogenic bacteria such as* E. faecalis, S. salivarius, Streptococcus sobrinus, Streptococcus sanguinis, S. mitis, S. mutans, *and* Lactobacillus casei *[59]. The essential oil displayed moderate antibacterial activity against* E. faecalis *(MIC = 250 *μ*g/mL) and* S. salivarius *(MIC = 250 *μ*g/mL). Meanwhile,* S. sobrinus* (MIC = 62.5 *μ*g/mL),* S. sanguinis* (MIC = 62.5 *μ*g/mL),* S. mitis* (MIC = 31.25 *μ*g/mL), and* Lactobacillus casei* (MIC = 31.25 *μ*g/mL) were significantly inhibited. Interestingly, the MIC value for* S. mutans* was found to be 3.9 *μ*g/mL. In another study, the essential oil of* Fortunella margarita* was shown to inhibit* Streptococcus faecalis* and* P. aeruginosa* significantly with inhibitory zones of 30 mm and 28 mm, respectively. In addition, moderate activity was observed for* B. subtilis, S. aureus, Sarcina lutea, *and* E. coli *with inhibitory zones ranging from 20 to 25 mm [36]. Similarly,* Achillea fragrantissima* essential oil was effective against* S. aureus*,* S. epidermidis*, and* E. coli* with the highest inhibition zone of 26 mm, 16 mm, and 16 mm, respectively [19]. In a study by Radaelli et al. [127], a major food-borne disease-causing agent,* C. perfringens*, was inhibited by essential oils of Brazilian MAPs such as basil, rosemary, marjoram, peppermint, thyme, and* Pimpinella anisum* (anise). The MIC values were 1.25 mg/mL for thyme, 5.0 mg/mL for marjoram and basil, and 10 mg/mL for peppermint, rosemary, and anise. Mahmoud et al. [128] have shown the antimicrobial potential of 11 essential oils against all the tested microbes (*S. aureus*,* E. coli, P. aeruginosa*, and* K. pneumoniae*). Onion oil exhibited good antibacterial activity (MIC = 12 *μ*g/mL) against* S. aureus*. Chamomile (*Anthemis nobilis*) oil showed the best activity against* P. aeruginosa* (MIC = 5.1 *μ*g/mL). Origanum and chamomile oils showed the highest antibacterial activity (MIC  7.2, 7.5, and  7.7 *μ*g/mL) against* E. coli. *Origanum and ivy (*Dolichos lablab*) oils were effective against* K. pneumoniae* with MIC values of 6.2 and 6.5 *μ*g/mL, respectively. More recent studies have revealed that essential oils of* Eucalyptus globulus, Matricaria chamomilla, Termitomyces schimperi, *and* R. officinalis *possess antimicrobial activity against* S. aureus, S. pyogenes, S. typhi, Shigella *spp*., E. coli, *and* P. aeruginosa *[129]. The essential oil of* Termitomyces schimperi* showed MIC values of <15.75 mg/mL for most of the tested bacteria, whereas other essential oils exhibited MIC values of 15.75–36.33 mg/mL against tested bacteria.

### 3.2. Antifungal Effects of Essential Oils

The essential oils and their constituents have been used against a broad range of fungal pathogens.  Table 2 summarizes various essential oils, their chemical compositions, and their antifungal activity against human pathogens. The essentials oils extracts from many plants such as basil, citrus, fennel, lemon grass, oregano, rosemary, and thyme have shown considerable antifungal activity against a wide range of fungal pathogens [65]. Arora and Kaur [120] observed the antimicrobial activity of essential oils extracted from spices against fungal pathogens. They found that garlic and clove extracts inhibited the growth of* Candida acutus, C. albicans, C. apicola, C. catenulata. C. inconspicua, C. tropicalis, Rhodotorula rubra, Saccharomyces cerevisiae, *and* Trigonopsis variabilis*. Similarly, Grohs and Kunz [29] investigated mixtures of ground spices and demonstrated their efficacy against the* C. lipolytica*. According to the report of Ultee and Smid [55], oregano and thyme essential oils were some of the best inhibitors of fungal pathogens, because of the phenolic compounds (carvacrol and thymol) as main constituents, which disrupt fungal cell membranes. Likewise, Delaquis and Mazza [130] reported the antimicrobial effects of isothiocyanate isolated from the essential oils of onion and garlic plants. They stated that isothiocyanates may inactivate the extracellular enzymes through oxidative cleavage of disulfide bonds. Isothiocyanate was effective against* Botrytis, Fusarium, Penicillium*, and* Cladosporium* species. The antifungal activity of essential oils and their derivatives on the cell viability, mycelium growth, and mycotoxin-producing ability of molds has been studied [131]. It was concluded that, among all the tested essential oils, clove, cinnamon, and oregano essential oils were effective against* Aspergillus parasiticus* and* Fusarium moniliforme*. The oil of* Origanum vulgare* was efficient at inhibiting* C. albicans*,* Aspergillus niger*,* Microsporum gypseum*,* Microsporum canis, Arthroderma cajetani, Trichophyton violaceum*,* Trichophyton mentagrophytes*,* Epidermophyton floccosum*,* T. rubrum*, and* Trichophyton tonsurans* [52, 56]. The MIC values ranged from 0.625 to 10.0 mg/mL against all the tested microbes. The essential oils of* Lippia sidoides*,* Rosmarinus officinalis*,* Salvia sclarea*, and* Momordica charantia* were shown to inhibit* C. albicans* effectively [39, 44, 65]. Clove essential oil showed an MIC value of 0.125 and 0.062% (v/v) against* C. albicans* and* A. niger*, respectively. Rosemary essential oil exhibited MIC values of 0.25 and 1.0% (v/v) against* C. albicans* and* A. niger*, respectively [65]. Thymol and carvacrol effectively inhibited* C. albicans* with inhibition zones of 10.6 and 9 mm, respectively [39].

Similarly, Jirovetz et al. [70] analyzed the antifungal activity of* Coriandrum sativum* oil against* Candida* species. The essential oil obtained from leaves and flowers of the* Ocimum *sp. showed considerable antifungal potential against* C. albicans, C. tropicalis, C. glabrata, Penicillium notatum, Rhizopus stolonifer,* and* Mucor mucedo* [27, 47, 48]. Likewise,* Myrtus communis* oil also inhibited* C. albicans, Aspergillus flavus,* and* Fusarium culmorum* [45, 85, 86]. The essential oils of thyme and clove completely inhibited the mycelial growth of* A. flavus* when 3 *μ*L of oil was added to the Petri-dish [86]. The MIC values of* M. communis* oil were found to be 50 *μ*L/mL for* A. flavus* and 30 *μ*L/mL for* A. ochraceus and F. culmorum* [45]. In addition, Bouzabata et al. [87] analyzed the antifungal activity of* M. communis* oil against* E. floccosum*,* Microsporum canis*,* Trichophyton rubrum *(dermatophytes), and* Cryptococcus neoformans *(yeast). An MIC value of 0.64 mg/mL was found to be lethal for* M. canis*,* T. rubrum,* and* E. floccosum*. However,* Candida* sp. and* Aspergillus* sp. strains were relatively less inhibited, with MIC values of 1.25 mg/mL and 5 mg/mL, respectively. Tea tree oil* (Melaleuca alternifolia)* was effective against many fungal pathogens such as* Alternaria *spp.,* A. flavus, A. fumigates, A. niger, Blastoschizomyces capitatus, C. albicans, C. glabrata, C. parapsilosis, C. tropicalis, Cladosporium *spp.,* Cryptococcus neoformans, E. floccosum, Fusarium *spp*., Malassezia furfur, Malassezia sympodialis, Microsporum canis, M. gypseum, Penicillium *spp.,* Rhodotorula rubra, S. cerevisiae, T. mentagrophytes, T. rubrum, T. tonsurans, *and* Trichosporon *sp. [81]. The essential oil of* Salvia sclarea*, a medicinal plant, contained 56.88% linalyl acetate, 20.75% linalool, 5.08% germacrene D, and 3.41%  *β*-caryophyllene as the chief chemical compounds. The essential oil and the pure compounds (linalyl acetate and linalool) were shown to possess antifungal properties against* C. albicans, C. tropicalis, C. krusei, C. glabrata, *and* C. parapsilosis* [84]. The antifungal activity of* Pogostemon cablin* oil against* Aspergillus* species and* C. albicans* has been reported by many authors [1, 90, 91]. MIC values of 0.064 mg/mL (cinnamon oil) and 0.032 mg/mL (pogostemon oil) for* C. albicans*, 0.129 mg/mL (cinnamon oil) and 0.064 mg/mL (pogostemon oil) for* C. tropicalis*, and 0.129 mg/mL (cinnamon oil) and 0.064 mg/mL (pogostemon oil) for* C. krusei* were observed [90]. The essential oils of* Mentha pulegium *and* M. suaveolens* were efficient at inhibiting fungal species such as* S. cerevisiae, Kloeckera apiculata, Candida zemplinina, Metschnikowia pulcherrima, *and* Tetrapisispora phaffii* [41]. The essential oil of* M. insularis* showed the highest activity against* Staphylococcus xylosus *with an MIC value of 3.6 *μ*L/mL. Moreover, Venturi et al. [82] reported the antifungal action of the essential oils extracted from* Glechon spathulata* and* G. marifolia* against the dermatophytic fungi* Trichophyton rubrum* and* Epidermophyton floccosum*. The MIC values ranged from 10 to 83 mg/mL against* T. rubrum* and 83 to 500 mg/mL against* E. floccosum*. The essential oil of* Daucus littoralis* was effective against* C. albicans* with the MIC value ranging from 20 to 40 *μ*L/mL [80]. Seed essential oil of* Nigella sativa* was shown to possess activity against* A.  flavus*,* F*. * moniliforme*,* Fusarium graminearum, *and* Penicillium viridicatum *[46]. This oil was very effective and showed up to 90% zone inhibition against* F. moniliforme.* Moreover, the dermatophytic fungus* T. rubrum* was repressed by the essential oil of* J. excelsa* and the MIC value was observed to be 128 mg/mL [38].

More recently, eugenol (an essential oil compound from clove) was shown to cause permanent damage to the cells of* C. albicans* and was considered to be an efficient antifungal agent. The MIC value of eugenol was found to be 1.0% v/v [92]. Beatovic et al. [57] have reported its antifungal potential against* Ocimum basilicum*,* Aspergillus ochraceus, A. versicolor, A. niger, A. fumigates, Trichoderma viride, *and* P. funiculosum.* Similarly, the inhibitory potential of* Aegle marmelos* oil against* C. albicans, A. niger, *and* F. oxysporum *was demonstrated. The essential oils extracted from* Eremanthus erythropappus*,* P. barbatus, *and* P. amboinicus* were shown to inhibit the growth of* C. albicans, Cryptococcus gattii, Cryptococcus neoformans,* and* S. cerevisiae* [33]. Papajani et al. [88] have reported the antifungal activity of rosemary essential oil against dermatophytes such as* A. cajetani*,* E. floccosum*,* M. gypseum*,* M. canis*,* T. violaceum*,* T. mentagrophytes*,* T. rubrum*, and* T. tonsurans* and phytopathogens such as* Botrytis cinerea* and* Pleomorphomonas oryzae*. According to them, concentration below 20 *μ*g/mL was not effective and they suggested the use of concentrations above 100 *μ*g/ml for better antifungal activity. The essential oil of* Fortunella margarita* exhibited activity against* A. niger *and* C. albicans* with a zone of inhibition of more than 30 mm [36]. In a recent study by Souza et al. [89], the essential oil of* Pelargonium graveolens* showed effective inhibitory potential against* C. tropicalis*, a pathogen resistant to clinically used antifungal agents. The essential oil of* P. graveolens* was found to be rich in geraniol and linalool. Four common essential oils of MAPs including litsea* (Litsea cubeba)*, oregano, marjoram* (Origanum majorana *L.), thymus, and their mixtures showed varied levels of antifungal activity against* C. albicans*,* C. tropicalis*,* C. krusei*,* C. guilliermondii*,* C. parapsilosis,* and* S. cerevisiae* [132]. More recently, the essential oils obtained from* E. globulus, M. chamomilla, T. schimperi, *and* R. officinalis* demonstrated effective antifungal activity against* Trichophyton *spp. and* Aspergillus *spp. [129].

### 3.3. Antiviral Effects of Essential Oils

Plant-based products and bioactive pure compounds may be a new source of antiviral drugs, as natural products have inherently high chemical diversity. Viral diseases are still a major problem for human health worldwide. So far, only a limited number of drugs are effective against many of these viruses, which has prompted research into finding new antiviral lead molecules. From our literature survey, it is evident that many essential oils possess antiviral properties against many DNA and RNA viruses, such as herpes simplex virus type 1 (HSV-1) and type 2 (HSV-2), dengue virus type 2, Junin virus, influenza virus adenovirus type 3, poliovirus, and coxsackievirus B1 [96, 105, 114, 133, 134].

The antiviral activities of essential oils of major MAPs along with their constituents are detailed in Table 3. The oregano and clove essential oils also exhibited strong antiviral activity against several nonenveloped RNA and DNA viruses such as adenovirus type 3, poliovirus, and coxsackievirus B1 [133, 134]. The replication capability of HSV-1 virus could be repressed by various essential oils under in vitro experimental conditions [135–137]. HSV-1 is the cause of common viral infections in humans, such as herpetic keratitis, herpetic encephalitis, mucocutaneous herpes infections, and neonatal herpes. Studies on the essential oils of* Artemisia arborescens, Glechon spathulata*, and* Glechon marifolia* found that they strongly suppressed HSV-1 [82, 93, 138].* Melissa officinalis* essential oils have major constituents, namely, citral and citronellal, which could inhibit the replication of HSV-2 [96, 133, 137]. Likewise, the antiherpes activities of Australian tea tree oil, eucalyptus oil, and thyme oil have been previously reported [93, 135–138]. The major chemical constituent *α*-caryophyllene, which occurs in many essential oils of medicinal plants, is considered to be the best antiviral agent [135].

Likewise, several phenylpropanoids and sesquiterpenes including eugenol, trans-anethole, *β*-eudesmol, *β*-caryophyllene, and farnesol, which are present in essential oils, also have antiviral properties against HSV [135]. Similarly, another major compound of essential oils, eugenol, showed virucidal activity against human herpesvirus [137, 139]. Some triterpenes and sesquiterpenes also possess antiviral activity against different herpesviruses and rhinovirus [140–143]. García et al. [144] reported the antiviral activity of* Artemisia douglasiana* and* Eupatorium patens* essential oils against the dengue virus. In addition,* Lippia junelliana *and* Lippia turbinate* essential oils showed activity against the Junin virus. Anti-influenza A (H2N2) activity was exhibited by the essential oil compounds of* Pogostemon cablin* [1, 97–99] and the antiviral property of the essential oils obtained from fruits and leaves of* Fortunella margarita* exhibited potential activity against avian influenza A virus (H5N1) [94]. Roy et al. [100] indicated the potential antiviral activity of* Trachyspermum ammi* oil against Japanese encephalitis virus (JEV). Similarly, Zeedan et al. [19] reported the antiviral activity of* Achillea fragrantissima* against the ORF virus (a parapox virus). More recently, Pourghanbari et al. [145] evaluated in vitro antiviral activity of* M. officinalis* (lemon balm) essential oil and oseltamivir and their synergistic effect on avian influenza virus (AIV) subtype H9N2. They found that various concentrations of lemon balm essential oil suppressed influenza virus replication. However, it had improved efficacy when coadministered with the antiviral agent oseltamivir. Essential oils obtained from Colombian MAPs such as* Lepechinia salviifolia*,* Minthostachys mollis*,* Hyptis mutabilis*,* Lepechinia vulcanicola,* and* Ocimum campechianum* were reported to possess antiviral activity against human herpes viruses types 1 and 2 [95]. They also reported that these essential oils inhibit viral activity during their early stages of infection. Thus, plant-based essential oils could be used as antiviral agents against several viral diseases in humans and have the potential to be used as alternatives to synthetic antiviral drugs.

## 4. Mechanism of Antimicrobial Action of Essential Oils against Human Pathogens

**Figure 2:**
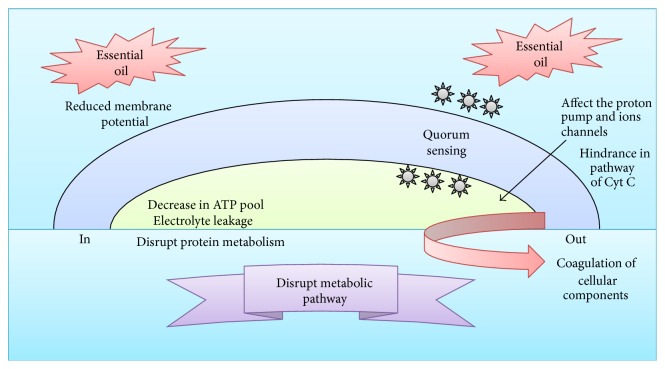
Antimicrobial mechanisms of essential oils on microbes.

MAPs contain several types of chemical constituents that have antimicrobial properties. These are synthesized to protect the plants from microbial pathogens. The antimicrobial properties of essential oils mainly depend on their chemical constituents and the quantity of the major single compounds [15]. These chemical compounds are secreted through a series of molecular interactions under specific biotic/abiotic stress conditions [15, 146]. Each compound may exhibit a different mechanism of action against microbes. Overall, the mechanism of antibacterial action is mediated by a series of biochemical reactions in the bacterial cell, which are dependent on the type of chemical constituents present in the essential oil [15, 110]. Moreover, the antibacterial activity of essential oils also differs because of different bacterial architecture, such as Gram-positive and Gram-negative bacteria, which differ in their cell membrane compositions [83, 114]. In the following sections, the mechanism of antimicrobial activities of essential oils is described with reference to the available literature. The possible antimicrobial actions of essential oils are illustrated in Figure 2.

### 4.1. Action against Bacterial Pathogens

Various mechanisms of antibacterial activity of essential oils have been proposed. Essential oils primarily destabilize the cellular architecture, leading to the breakdown of membrane integrity and increased permeability, which disrupts many cellular activities, including energy production (membrane-coupled), membrane transport, and other metabolic regulatory functions. The disruption of the cell membrane by essential oils may assist various vital processes such as energy conversion processes, nutrient processing, the synthesis of structural macromolecules, and the secretion of growth regulators [66]. The essential oils may affect both the external envelope of the cell and the cytoplasm [15, 114]. Owing to their lipophilic nature, essential oils are easily penetrable through the bacterial cell membranes. Essential oils of various MAPs were reported to cause increased bacterial cell membrane permeability leading to the leakage of cellular components and loss of ions [66, 114, 147]. The antibacterial effect of essential oils is also linked to reduced membrane potentials, the disruption of proton pumps, and the depletion of the ATP [148]. This alteration in the cell organization may cause a cascade effect, resulting in other cell organelles being affected [43]. Likewise, Cox et al. [149, 150] have demonstrated that tea tree oil inhibits the growth of* S. aureus* and* E. coli* by altering cell permeability, increasing the leakage of intracellular K+ ions and disturbing cell respiration. The essential oils pass through the cell wall and cytoplasmic membrane, which may disrupt the arrangement of dissimilar fatty acids, phospholipids bilayers, and polysaccharides molecules [114, 147, 151]. All these events may be responsible for the coagulation of inner cellular components in the cytoplasm and break down of the bonds between the lipid and protein layers [110].

In some cases, the pure compounds of essential oils exhibit higher antibacterial activity compared to the essential oil. The antibacterial effect of essential oil constituents such as thymol, menthol, and linalyl acetate is because of a perturbation of the lipid fractions of bacterial plasma membranes [152]. This may affect the permeability of the membrane and induce leakage of intracellular materials. Carvacrol is a hydrophobic compound that influences cell membranes by altering the composition of fatty acids, which then affects the membrane fluidity and permeability [16]. However, its exact mechanism of action is still unclear. It was reported that carvacrol significantly depleted the internal ATP pool of bacterial cells [16, 153]. In another study, carvacrol induced the leakage and loss of ATP from bacterial cells [154]. Likewise, the compounds methyl carvacrol, menthol, citronellol, and thymol also cause an enlargement of the cell membrane that leads to passive diffusion of ions between the expanded phospholipids [16, 147, 153, 154]. Another effect of essential oils on cell membranes is the inhibition of toxin secretion. Ultee and Smid [55] reported that exposure of* B. cereus *to carvacrol resulted in the inhibition of toxin production, and application of oregano essential oil completely abolished the enterotoxin production of* S. aureus. *Thus, the secretion of toxins may be prevented by modifications in the bacterial membrane due to the influence of the essential oil compounds on the trans-membrane transport process in the plasma membrane, which limits the release of toxins to the external environment [155]. Another mechanism of action is by* trans*-cinnamaldehyde, which enters the periplasm of the cell and disrupts cellular functions [16, 156]. Moreover,* p-*cymene has a greater affinity towards bacterial cell membranes and thus may disturb the membrane integrity [16, 157]. The outer membrane proteins are also affected by essential oil components. For example, carvacrol can disturb the insertion and folding of proteins such as DnaK and GroEL [16, 110]. Carvacrol can also inhibit the synthesis of flagellin, a microbial protein required for bacterial motility [16]. The phenylpropene, eugenol, also exhibits activity by modifying the fatty acid outline to alter the cytoplasmic membrane of different bacteria. In addition, it can destroy various bacterial enzymes such as ATPase, amylase, histidine carboxylase, and proteases [158, 159]. Likewise, cinnamaldehyde was reported to inhibit ATPase enzymes and disrupt the outer cell membrane [160]. Other studies have found that vanillin exhibited antimicrobial activity by obstructing the pathways of bacterial respiration and disrupting the flux of K+ ions and pH gradient [161]. Similarly, carveol, citronellal, and carvone essential oils were shown to modify hydrophobicity and disrupt membrane integrity, leading to the leakage of K^+^ions [162]. Some essential oils can inhibit the cell-cell communication quorum sensing network mediated by various bacterial signal molecules [163]. The efficacy of the antibacterial effect of essential oils or their individual compounds may differ from one microbe to another. Hence, elucidation on the exact mechanisms of action of each essential oil and their components is required, including further study on the numerous microbial strains/species. Furthermore, detailed study on the components of essential oils would be helpful to improve our understanding of their mechanism of antimicrobial activity.

### 4.2. Action against the Fungal Pathogens

The antifungal actions of essential oils are similar to that of previously explained antibacterial mechanisms. Generally, exposure of essential oils leads to the coagulation of the cellular components because of irreversible cell membrane damage. In yeast cells, essential oils establish a membrane potential across the cell membrane and disrupt the production of ATP, which leads to cell membrane damage [85]. The essential oils have the ability to penetrate and disrupt the fungal cell wall and cytoplasmic membranes through a permeabilization process, which leads to the disintegration of mitochondrial membranes. This is caused by alterations in the flow of electrons inside the electron transport system (ETS) pathway. This may also damage the lipids, proteins, and nucleic acid contents of cells infected by the fungal pathogens [164]. The essential oils could also disrupt the depolarization of the mitochondrial membranes by affecting ions channels, especially Ca^2+^ions, proton pumps, and ATP pools, and therefore decrease the membrane potential. This change in the fluidity of membranes may cause electrolyte leakage and hinder cytochrome C pathways, proteins metabolism, and calcium ion concentrations. Therefore, the permeabilization of inner and outer mitochondrial membranes may result in the cell apoptosis or necrosis leading to cell death [165].

### 4.3. Actions against the Viruses

At present, various essential oils may be a promising alternative against viral infections [105]. However, the detailed understanding on the antiviral action of essential oils still requires more research. Some of the reported mechanisms of action of essential oils are reported in this section. Essential oils might interfere with virion envelopment, designed for entry into host cells. For instance, the sesquiterpene triptofordin C-2 was reported to suppress the synthesis of viral proteins and inhibit the early gene expression process of the HSV-1 virus [141]. Schnitzler et al. [135] investigated the antiviral activity of star anise essential oil as well as compounds such as eugenol,* trans*-anethole, farnesol, *β*-eudesmol, *β*-caryophyllene, and *β*-caryophyllene oxide against HSV-1. They found the direct inactivation of HSV-1 particles, which is also reported in another study where eugenol was used [141]. Moreover, eugenol directly inactivates the growth of the herpes virus [139], whereas isoborneol (monoterpene) affected the glycosylation process of viral proteins, which inhibited the growth of HSV-1 [166]. Similarly, essential oils of ginger, thyme, hyssop, and sandalwood were able to inhibit acyclovir-resistant HSV-1 [136]. Possible mechanisms of action include the inhibition of virus replication by hindering cellular DNA polymerase and alteration in phenylpropanoid pathways. Furthermore, sesquiterpenes are known to inhibit cytomegalovirus (CMV) early gene expression [142]. According to Pourghanbari et al. [145], the essential oil of lemon balm inhibits influenza virus replication at different replication cycles by directly interacting with the virus particles.

## 5. Conclusion and Future Prospects

The essential oils extracted from various MAPs possess strong antimicrobial activity against various bacterial, fungal, and viral pathogens. The reactivity of essential oils depends upon the nature of their functional groups and orientation. Essential oils are considered to be potent against a diverse range of pathogens. Essential oils may disrupt the cell membrane of the targeted pathogens by increasing membrane permeability, inducing leakage of vital intracellular constituents, and interrupting the cellular metabolism and enzyme kinetics of the targeted pathogens. The present study reveals more information on in vitro research studies of essential oils; however, more efforts are required to conduct clinical trials in the future. Most of these antimicrobial studies using essential oils have failed to provide definite information on their chemical nature as well as their mechanisms of action. This poses ambiguity on the reproducibility and accuracy of their discoveries. Therefore, further research should focus on exploring the molecular mechanisms of essential oils and their individual chemical compounds. Biopharmaceutical industries are in need of ecofriendly alternative drug molecules to treat diseases associated with microbial pathogens and body metabolism. Thus, essential oils of MAPs might be a prospective source of alternative antimicrobial agents and may play an important role in the discovery of new drugs for the treatment of a wide range of pathogenic microorganisms in the near future.

## Figures and Tables

**Table 1:** Chemical composition of various essential oils and their antibacterial activity against human pathogens.

MAPs	Part used	Major chemical compounds	Inhibited microorganisms	References
*Achillea clavennae*	Leaves and flowers	Camphor, myrcene, 1,8-cineole, *β*-caryophyllene, linalool, geranyl acetate	*Klebsiella pneumonia*, *Streptococcus pneumonia*, *Haemophilus influenzae*, *Pseudomonas aeruginosa*	[18]

*Achillea fragrantissima*	Aerial parts	Yomogi alcohol, 1,8-cineole, artemisia alcohol, thujone	*Staphylococcus aureus*, *Staphylococcus epidermidis*, *Escherichia coli*	[19]

*Achillea ligustica*	Aerial parts	Viridiflorol, terpinen-4-ol	*Streptococcus mutans*	[20]

*Artemisia absinthium*	Aerial parts	Myrcene, *trans*-thujone, *trans*-sabinyl acetate	*E. coli*, *S. aureus*, *Staphylococcus epidermidis*	[21]

*Artemisia biennis*	Aerial parts	(*Z*)-Beta-ocimene, (*E*)-beta-farnesene, acetylenes, (*Z*)- and (*E*)-En-yn-dicycloethers	*E. coli*, *S. aureus*, *S. epidermidis*	[21]

*Artemisia cana*	Aerial parts	Santolina triene, alpha-pinene, camphene	*E. coli*, *S. aureus*, *S. epidermidis*	[21]

*Artemisia dracunculus*	Aerial parts	Methylchavicol, methyl eugenol, beta-phellandrene, terpinolene	*E. coli*, *S. aureus*, *S. epidermidis*, *Brochothrix thermosphacta*, *Listeria innocua*, *L. monocytogenes*, *Pseudomonas putida*, *Shewanella putrefaciens*	[21, 22]

*Artemisia longifolia*	Aerial parts	Alpha-pinene, camphene, 1,8-cineole	*E. coli*, *S. aureus*, *S. epidermidis*	[21]

*Artemisia frigida*	Aerial parts	1,8-Cineole, methylchavicol, camphor	*E. coli*, *S. aureus*, *S. epidermidis*	[21]

*Cinnamomum zeylancium*	Bark, leaves	Cinnamaldehyde	Enterobacteriaceae, *S. aureus, Streptococcus pyogenes*, *S. pneumoniae*, *Enterococcus faecalis*, *E. faecium*, *Bacillus cereus*, *Acinetobacter lwoffii*, *Enterobacter aerogenes*, *E. coli*, *Klebsiella pneumoniae*, *Proteus mirabilis*, *P. aeruginosa*, *Salmonella typhimurium*, *Clostridium perfringens*, *Mycobacterium smegmatis*	[23, 24]

*Copaifera officinalis*	Essential oil	*β*-Caryophyllene, *β*-bisabolene, germacrene B, *α*-copaene, germacrene D, *α*-humulene, *δ*-cadinene	*S. aureus*, *E. coli*	[25]

*Coriandrum sativum*	Leaves	2E-Decenal, decanal, 2E-decen-1-ol, n-decanol	*S. aureus*, *Bacillus *spp., *E. coli*, *Salmonella typhi*, *K. pneumonia*, *Proteus mirabilis*, *P. aeruginosa*	[26, 27]

*Cuminum cyminum*	Leaves	*γ*-Terpin-7-al, *γ*-terpinene, *β*-pinene, cuminaldehyde	*S. typhimurium*, *E. coli*	[28]

*Cymbopogon citratus*	Fruit	Ethanolic compounds	Enterobacteriaceae,* S. aureus*	[29]

*Cymbopogon nardus*	Leaves, stems	Δ2-Carene, beta-citronellal	*Brochothrix thermosphacta*, *E. coli*, *Listeria innocua*, *L. monocytogenes*, *P. putida*, *S. typhimurium*, *S. putrefaciens*	[22]

*Cyperus longus*	Arial part	*β*-Himachalene, *α*-humulene, *γ*-himachalene	*S. aureus*, *L. monocytogenes*, *L. monocytogenes*, *E. faecium*, *S. Enteritidis*, *E. coli*, *P. aeruginosa*	[30]

*Daucus littoralis*	Leaves, stems, roots, flowers, fruits	Germacrene D, acorenone B	*S. aureus*, *E. coli*	[31]

*Dracocephalum foetidum*	Leaves	n-Mentha-1,8-dien-10-al, limonene, geranial, neral	*B. subtilis*, *S. aureus*, *M.luteus*, *E. hirae*, *S. mutans*, *E. coli*	[32]

*Eremanthus erythropapps*	Leaves	(Z)-Caryophyllene, germacrene D, viridiflorol, *p*-cymene, *γ*-terpinene	*S. epidermidis*	[33]

*Eugenia caryophyllata*	Flower buds	Phenylpropanoids such as carvacrol, thymol, eugenol, cinnamaldehyde	*S. epidermidis*	[34]

*Euphrasia rostkoviana*	Essential oil	n-Hexadecanoic acid, thymol, myristic acid, linalool	*E. faecalis*, *E. coli*, *K. pneumoniae*, *S. aureus*, *S. epidermidis*, *P. aeruginosa*	[35]

*Foeniculum vulgare*	Leaves	Trans-anethole, methylchavicol, limonene	*S. typhimurium*, *E. coli*	[28]

*Fortunella margarita*	Leaves	Gurjunene, eudesmol, muurolene	*B. subtilis*, *S. aureus*, *Sarcina luta*, *S. faecalis*, *E. coli*, *K. pneumonia*, *P. aeruginosa*	[36]

*Juniperus phoenicea*	Arial part	*α*-Pinene, *β*-phellandrene, *α*-terpinyl acetate	*S. aureus*, *L. monocytogenes*, *L. monocytogenes*, *E. faecium*, *S. Enteritidis*, *E. coli*, *P. aeruginosa*	[30]

*Laurus nobilis*	Arial part	Eucalyptol (1,8-cineole), linalool	*Mycobacterium smegmatis*, *E. coli*	[37]

Lavandula x intermedia “Provence” (Blue Lavandin) (a cross between *L. angustifolia*, *L. Latifolia*)	Arial part	Camphor, eucalyptol (1,8-cineole), linalool, *β*-pinene, *α*-pinene	*M. smegmatis*, *E. coli*	[37]

*Juniperus excelsa*	Leaves and twigs	*α*-Pinene, *α*-cedrol, *δ*-car-3-ene	*S. aureus *	[38]

*Lippia sidoides*	Leaves	Thymol and carvacrol	*S. mutans*, *S. sanguis*, *S. salivarius*, *S. mitis*	[39]

*Mentha piperita*	Arial part		*S. aureus*, *S. typhimurium, V. parahaemolyticus*	[40]

*Mentha pulegium*	Arial part	Piperitone, piperitenone, *α*-terpineol, pulegone	*S. aureus*, *S. epidermidis*, *B. cereus*, *L. monocytogenes*, *E. coli*, *S. typhimurium*, *V. cholera*, *L. monocytogenes*, *E. faecium*, *S. Enteritidis*	[30]

*Mentha suaveolens*	Arial part	Pulegone, piperitone, cis-cis-p-menthenolide, limonene germacrene	*Lactococcus lactis subsp*. *Lactis*, *S. xylosus*	[41]

*Melaleuca alternifolia *(tea tree oil)	Essential oil	Terpinen-4-ol, 1,8-cineole, *γ*-terpinene, *α*-terpinene, terpinolene	*E. coli*, *S. aureus*, *S. epidermidis*, *E. faecalis*, *P. aeruginosa*, *M. avium*, *H. influenzae*, *S. pyogenes*, *S. pneumonia*	[42, 43]

*Momordica charantia*	Seed	Trans-nerolidol, apiole, cis-dihydrocarve,ol germacrene D	*E. coli*, *S. aureus*	[44]

*Myrtus communis *	Leaves	Eugenol, *α*-terpineol, *γ*-terpinene	*S. aureus*, *L. monocytogenes*, *E. durans*, *Salmonella Typhi*, *E. coli*, *B. subtilis*, *M. tuberculosis, P. aeruginosa*, *K. pneumonia, M. avium *subsp. *paratuberculosis*, *E. cloacae*	[30, 45]

*Nigella sativa*	Seeds	Thymoquinone, *p*-cymene, *α*-thujene, thymohydroquinone, longifolene	*S. aureus*, *B. cereus*, *E. coli*, *P. aeruginosa*	[46]

*Ocimum gratissimum*	Leaves	Eugenol, methyl eugenol, cis-ocimene, trans-ocimene, *α*-pinene, camphor	*S. aureus, Bacillus *spp. *E. coli*, *P. aeruginosa*,* S. typhi*, *K. pneumoniae*, *P. mirabilis*, *E. cloacae*	[26, 47]

*Ocimum kilimandscharicum*	Flowers and leaves	Eugenol, borneol, linalool, methyl eugenol	*B. subtilis*, *S. aureus*, *Citrobacter youngae*,* E. coli*, *Klebsiella *spp., *Micrococcus *spp., *Proteus *spp., *Pseudomonas *spp., *Salmonella spp.*	[48]

*Origanum vulgare*	Leaves, Arial part	Carvacrol, thymol, *γ*-terpinene, *trans*-sabinene hydrate, *cis*-piperitol, borneol, terpinen-4-ol, linalool	*Clostridium botulinum, C. perfringens*, *L. monocytogenes*,* E. coli*, S*. choleraesuis*, *S. typhimurium*, *S. aureus*, *B. subtilis*, *Pseudomonas aeruginosa*, *Shigella sonnei*,* Sarcina lutea*, *M. flavus*, *K. pneumoniae*, *K. oxytoca*	[49–56]

*Ocimum basilicum*	Leaves, stems	*γ*-Terpinene, methylchavicol	*Brochothrix thermosphacta*, *E. coli*, *L. innocua*, *L. monocytogenes*, *P. putida*, *S. typhimurium*, *S. putrefaciens*, *M. flavus*	[22, 57]

*Petroselinum sativum*	Leaves, stems	Myristicin, apiol, 1,2,3,4-tetramethoxy-5-(2-propenyl)- benzene	*B. thermosphacta*, *E. coli*, *L. innocua*, *L. monocytogenes*, *P. putida*, *S. typhimurium*, *S. putrefaciens*	[22]

*Piper nigrum*	Essential oil	Limonene, *δ*-3-carene, *α*-pinene, *β*-caryophyllene, *β*-pinene, sabinene, *α*-felandeno, myrcene, para-cymene, linalool, terpinolene, *β*-selinene, 1,8 cineole, *α*-terpinene, *α*-humulene, *α*-copaene, eugenol, terpinen-4-ol, camphene, safrole	*S. aureus*, *E. coli*	[25]

*Pimpinella anisum*	Seed	Trans-anethole	*S. typhimurium*, *E. coli*	[58]

*Plectranthus barbatus *	Leaves	(Z)-Caryophyllene, germacrene D, viridiflorol, *p*-cymene, *γ*-terpinene	*S. epidermidis*	[4, 33]

*P. amboinicus*	Leaves	(Z)-Caryophyllene, germacrene D, viridiflorol, *p*-cymene, *γ*-terpinene	*S. epidermidis*	[4, 33]

*Plectranthus neochilus*	Leaves	*α*-Pinene, *β*-pinene, trans-caryophyllene, caryophyllene oxide	*E. faecalis*, *S. salivarius*, *S. sobrinus*, *S. sanguinis*, *S. mitis*, *L. casei*, *S. mutans*	[4, 59]

*Pogostemon cablin *	Leaves	Patchoulol, *δ*-guaieno; gurjunene-*α*, *α*-guaiene, aromadendrene, *β*-patchoulene	*K. pneumonia*, *H. pylori*, *E. coli*, *B. subtilis*, *S. aureus*, *P. aeruginosa*, *E. faecalis*	[1, 60–64]

*Rosmarinus officinalis*	Leaves, flower	Camphor, camphene, limonene, geraniol, myrcene, linalool benzoylacetate, linalool, *α*-pinene, *α*-terpinolene, bornyl acetate, borneol	*E. coli*, *S. typhimurium*, *B. cereus*, *Bacillus subtilis*, *S. aureus*, *S. agalactiae*, *S. epidermidis, S. aureus*, *P. vulgaris*, *P. aeruginosa*, *K. pneumonia*, *E. faecalis*, *B. thermosphacta*, *L. innocua*, *L. monocytogenes*, *P. putida*, *S. typhimurium*, *S. putrefaciens*, *M. smegmatis*	[22, 37, 65, 66]

*Satureja hortensis*	Arial part	Carvacrol, thymol, *γ*-terpinene	*C. botulinum*, *C. perfringens*,	[49]

*Salvia sclarea*	Arial part	Linalool, linalyl acetate, geranyl acetate, *β*- ocimene acetate, caryophyllene oxide	*S. aureus*, *S. agalactiae*, *S. epidermis*, *E. coli*, *Proteus vulgaris*, *P. aeruginosa*, *K. pneumonia*, *E. faecalis*, *B. pumilus*, *B. subtilis*, *S. typhimurium*	[67–70]

*Salvia officinalis*	Arial part	*α*-Thujone, camphor, 1,8-cineole, *α*-pinene	*S. aureus*, *P. stuartii*, *P. stuartii*, *E. coli*, *Shigella sonnei*,* Sarcina lutea*, *M. flavus*, *B. thermosphacta*, *E. coli*, *L. innocua*, *L. monocytogenes*	[17, 22, 70]

*Salvia lavandulifolia*	Essential oil	Camphor, *α*-thujone, beta-thujone, camphene, *α*-pinene, terpineol	*P. vulgaris*, *P. aeruginosa*, *K. pneumonia*, *E. faecalis*	[68, 70]

*Satureja cuneifolia*	Aerial parts	Carvacrol and *p*-cymene	*E. coli*, *Campylobacter jejuni*, *S. sonnei*, *S. aureus*, *L. monocytogenes*, *B. cereus*, *P. aeruginosa*, *S. enteritidis*	[71]

*Struchium sparganophora*	Leaves	*β*-Caryophyllene, germacrene A, *α*-humulene, germacrene D	*S. typhi*, *B. cereus*, *P. mirabilis*, *P. aeruginosa*, *B. subtilis*	[72]

*Syzygium aromaticum *	Leaves, flower bud	Eugenol, eugenylacetate	*P. aeruginosa*, Enterobacteriaceae	[22, 25]

*Syzygium cumini*	Leaves	*α*-Pinene, *β*-pinene, trans- caryophyllene, 1,3,6-octatriene, delta-3-carene, *α*-caryophyllene, *α*-limonene	*E. coli*, *S. aureus*, *P. aeruginosa*, *N. gonorrhoeae*, *B. subtilis*, *S. aureus*	[73]

*Trachyspermum ammi*	Seeds	—	*K. pneumoniae*, *E. coli*, *S. aureus*	[74]

*Thymus vulgaris*	Arial part	Thymol, linalool, carvacrol, 1,8-cineole, eugenol, camphor, camphene, *α*-pinene, borneol, *β*-pinene	*L. monocytogenes, E. coli,S. typhimurium*, *S. aureus*, *C. botulinum, C. perfringens*, *S. sonnei*,* S. lutea*, *M. flavus*, *B. thermosphacta*, *L. innocua*, *L. monocytogenes*, *P. putida*, *S. putrefaciens*	[22, 40, 49, 53, 54, 75, 76]

*Thymus zygis*	Essential oil	—	*S. choleraesuis*, *S. typhimurium*, *E. coli*	[50]

*Thymus mastichina*	Leaves, stems	m-Thymol, carvacrol, trans-caryophyllene	*B. thermosphacta*, *E. coli*, *L. innocua*, *L. monocytogenes*, *P. putida*, *S. typhimurium*, *S. putrefaciens*	[22]

*Thymus kotschyanus*	Arial part	Carvacrol, 1,8 cineole, thymol, borneol, E-caryophyllene	*S. aureus*, *S. epidermidis*, *B. cereus*, *E. coli*	[77]

*Thuja* sp. *(Thuja plicata*, *Thuja occidentalis)*	Essential oil	Alpha-thujone and beta-thujone	*P. aeruginosa*, *K. pneumoniae*, *S. aureus*, *E. coli*	[68]

*Verbena officinalis*	Arial part	Borneol, geranoil	*S. aureus*, *E. coli*, *S. typhimurium*, *L. monocytogenes*	[78]

*Warionia saharae*	Arial part	*β*-Eudesmol, trans-nerolidol, linalool, 1,8 cineole, camphor, *p*-cymene, terpinen-4-ol	*S. aureus*, *B. cereus, P. aeruginosa*, *E. coli*	[79]

**Table 2:** Chemical composition of various essential oils and their antifungal activity against human pathogens.

MAPs	Part used	Major chemical compounds	Inhibited microorganisms	References
*Aegle marmelos*	Leaves	*γ*-Cadinene, *δ*-carene, *α*-pinene	*Candida albicans*, *Aspergillus niger*, *Fusarium oxysporum*	[36]

*Artemisia biennis*	Aerial parts	(*Z*)-*β*-Ocimene, (*E*)-beta-farnesene, acetylenes, (*Z*)- and (*E*)-en-yn-dicycloethers	*Cryptococcus neoformans*, *Fonsecaea pedrosoi*, *A. niger*	[21]

*Cinnamomum zeylancium*	Bark, leaves	Cinnamaldehyde	*C. albicans*, *C. parapsilosis*, *C. krusei*	[23, 24]

*Coriandrum sativum*	Leaves	2E-Decenal, decanal, 2E-decen-1-ol, n-decanol	*C. albicans*	[26, 27]

*Daucus littoralis*	Leaves, stems, roots, flowers, fruits	Germacrene D, acorenone B	*C. albicans*	[80]

*Dracocephalum foetidum*	Leaves	n-Mentha-1,8-dien-10-al, limonene, geranial, neral	*C. albicans*	[32]

*Eremanthuserythropappus*	Leaves	(Z)-Caryophyllene, germacrene D, viridiflorol, *p*-cymene, *γ*-terpinene	*C. albicans*, *C. gattii*, *C. gattii*, *C. neoformans*, *S. cerevisiae*	[33]

*Euphrasia rostkoviana*	Essential oil	n-Hexadecanoic acid, thymol, myristic acid, linalool	*C. albicans*	[35]

*Feoniculum vulgare*	Seed	*Trans*-anethole, methylchavicol, limonene	*Alternaria alternata*, *F. oxysporum*, *A. flavus*	[81]

*Fortunella margarita*	Leaves	Gurjunene, eudesmol, muurolene	*A. niger*, *C. albicans*	[36]

*Glechon spathulata*	Leaves	*β*-Caryophyllene, bicyclogermacrene	*Trichophyton rubrum*, *Epidermophyton floccosum*	[82]

*Glechon marifolia*	Leaves	*β*-Caryophyllene, bicyclogermacrene	*T. rubrum*, *E. floccosum*	[82]

*Lippia sidoides*	Leaves	Thymol and carvacrol	*C. albicans*	[39]

*Melaleuca alternifolia* (tea tree oil)	Essential oil	Terpinen-4-ol, 1,8-cineole, *γ*-terpinene, *α*-terpinene, terpinolene	*Alternaria spp*. *A. flavus*, *A. fumigates*, *A. niger*, *Blastoschizomyces Capitatus*, *C. albicans*, *C. glabrata*, *C. parapsilosis*, *C. tropicalis*, *Cladosporium *spp., *C. neoformans*, *Epidermophyton floccosum*, *Fusarium *spp., *Malassezia furfur*, *Microsporum canis*, *M. sympodialis*, *M. gypseum*, *Penicillium *spp., *Rhodotorula rubra*, *Saccharomyces cerevisiae*,* Trichophyton mentagrophytes*, *T. rubrum*, *T. tonsurans*, *Trichosporon *spp.	[42, 81, 83, 84]

*Mentha pulegium*	Arial part	Piperitone, piperitenone, *α*-terpineol pulegone	*A. niger*, *C. albicans*, *C. zemplinina*, *Kloeckera apiculata*, *Metschnikowia pulcherrima, Tetrapisispora phaffii*	[30, 41]

*Momordica charantia*	Seed	Trans-nerolidol, apiole, cis-dihydrocarveol, germacrene D	*C. albicans*	[44]

*Myrtus communis *	Leaves	Eugenol, *α*-terpineol, *γ*-terpinene, *α*-caryophyllene	*C. albicans*, *A. flavus*	[45, 85–87]

*Nigella sativa*	Seeds	Thymoquinone, *p*-cymene, *α*-thujene, thymohydroquinone, longifolene	*A. flavus*, *Fusarium moniliforme*, *F. graminearum*, *P. viridicatum*	[46, 62]

Ocimum species (*Ocimum basilicum*, *Ocimum gratissimum*, *O. kilimandscharicum*, *O. lamiifolium*, *O. suave*)	Leaves, flower	Eugenol, methyl eugenol, cis-ocimene, trans-ocimene, *α*-pinene camphor	*C. albicans*, *C. tropicalis*, *C. glabrata*, *P. notatum*, *R. stolonifer*, *M. mucedo*, *A. ochraceus*, *A. versicolor*, *A. niger*, *A. fumigates*, *T. viride*, *P. funiculosum*	[26, 27, 47, 48, 57]

*Origanum vulgare*	Leaves, arial part	Carvacrol, thymol, *γ*-terpinene, *trans*-sabinene hydrate, *cis*-piperitol, borneol, terpinen-4-ol, linalool	*C. albicans*, *A. niger*, *M. gypseum*, *M. canis*, *A. cajetani*, *T. violaceum*, *T. mentagrophytes*, *E. floccosum*, *T. rubrum*, *T. tonsurans, phytopathogens B. cinerea and P. oryzae*	[52, 56, 88]

*Pelargonium graveolens*	Leaves	Citronellol, citronellyl formate, geraniol	*C. tropicalis*	[89]

*Plectranthus barbatus and P. amboinicus*	Leaves	(Z)-Caryophyllene, germacrene D, viridiflorol, *p*-cymene, *γ*-terpinene	*C. albicans*, *C. gattii*, *C. gattii*, *C. neoformans*, *S. cerevisiae*.	[4, 33]

*Pogostemon cablin *	Leaves	Patchoulol, *δ*-guaieno; gurjunene-*α*, *α*-guaiene, aromadendrene, *β*-patchoulene	Aspergillus species, *C. albicans*	[1, 90, 91]

*Rosmarinus officinalis*	Leaves	Camphor, camphene, limonene, geraniol, myrcene, linalool benzaylacetate, linalool, *α*-pinene, *α*-terpinolene, bornyl acetate, borneol	*C. albicans*,* M. gypseum*, *M. canis*, *A. cajetani*, *T. violaceum*, *T. mentagrophytes*, *E. floccosum*, *T. rubrum*, *T. tonsurans*, *phytopathogens B. cinerea*, *P. oryzae*	[65, 88]

*Salvia sclarea*	Arial part	Linalool, linalyl acetate, geranyl acetate, *β*- ocimene acetate, caryophyllene oxide	*C. albicans*, *C. tropicalis*, *C. krusei*, *C. glabrata*, *C. parapsilosis*	[39, 84]

*Syzygium aromaticum *	Leaves	Eugenol, eugenylacetate	*A. fumigatus*, *C. albicans*,* Candida *spp.	[25, 92]

**Table 3:** Chemical composition of various essential oils and their antiviral activity against human pathogens.

Plant	Part used	Chemical compounds	Inhibited microorganisms	References
*Achillea fragrantissima*	Aerial parts	2,5,5-Trimethyl-3,6-heptadien-2-ol, eucalyptol, artemisia alcohol, thujone	ORF virus (a parapox virus)	[19]

*Artemisia arborescens*	Aerial parts	*β*-Thujone, linalool, myrcene, carvacrol	Herpes simplex virus type 1 (HSV-1)	[93]

*Fortunella margarita*	Leaves	Gurjunene, eudesmol, muurolene	Avian influenza A virus (H5N1),	[94]

*Glechon spathulata *	Leaves	*β*-Caryophyllene, bicyclogermacrene	HSV-1	[82]

*Glechon marifolia*	Leaves	*β*-Caryophyllene, bicyclogermacrene	HSV-1	[82]

*Hyptis mutabilis*	Leaves	*α*-Phellandrene, *p*-cymene, E-caryophyllene	HSV-1	[95]

*Lepechinia salviifolia*	Leaves	Germacrene D	HSV-1	[95]

*Melissa officinalis*	Leaves	Myrcene, linalool, camphor, citronellal, *β*-caryophyllene, caryophyllene oxide, citral	HSV-2, avian influenza virus (AIV) subtype H9N2	[19, 96]

*Minthostachys mollis*	Leaves	*α*-Pinene, estragole	HSV-1	[95]

*Ocimum campechianum*	Leaves	Linalool, eugenol	HSV-1	[95]

*Pogostemon cablin *	Leaves	Patchoulol, *δ*-guaieno; gurjunene-*α*, *α*-guaiene, aromadendrene, *β*-patchoulene	Influenza A (H2N2) virus	[1, 97–99]

*Trachyspermum ammi*	Leaves	Thymol, *α*-pinene, *p*-cymene, limonene	Japanese encephalitis virus (JEV)	[100]
